# B-Cell Dysregulation in Idiopathic Nephrotic Syndrome: What We Know and What We Need to Discover

**DOI:** 10.3389/fimmu.2022.823204

**Published:** 2022-01-24

**Authors:** Manuela Colucci, Julie Oniszczuk, Marina Vivarelli, Vincent Audard

**Affiliations:** ^1^ Renal Diseases Research Unit, Genetics and Rare Diseases Research Area, Bambino Gesù Children’s Hospital, Istituto di Ricovero e Cura a Carattere Scientifico (IRCCS), Rome, Italy; ^2^ Assistance Publique des Hôpitaux de Paris, Hôpitaux Universitaires Henri-Mondor, Service de Néphrologie et Transplantation, Centre de Référence Maladie Rare “Syndrome Néphrotique Idiopathique”, Fédération Hospitalo-Universitaire, Innovative Therapy for Immune Disorders, Créteil, France; ^3^ Univ Paris Est Créteil, Institut National de la Santé et de la Recherche Médicale (INSERM) U955, Institut Mondor de Recherche Biomédicale (IMRB), Créteil, France; ^4^ Division of Nephrology, Department of Pediatric Subspecialties, Bambino Gesù Children’s Hospital, Istituto di Ricovero e Cura a Carattere Scientifico (IRCCS), Rome, Italy

**Keywords:** Idiopathic Nephrotic syndrome (INS), minimal change disease (MCD), focal segmental glomerulosclerosis (FSGS), B cells, antibodies, cytokines

## Abstract

The therapeutic efficacy of B-cell depletion by anti-CD20 treatment in pediatric and, more recently, in adult idiopathic nephrotic syndrome patients suggests a key role of B cells in the pathogenesis of the disease. However, their exact role is still unclear. B cells are able to secrete a large variety of antibodies that can protect against infections. However, B-cell dysregulation is well-established in a variety of autoimmune diseases. In parallel with their ability to produce antibodies, pathogenic B cells display altered effector functions by expressing activating surface molecules, which can strongly modify the immune homeostasis, or by producing specific cytokines, which can directly affect either podocyte structure and functions or modulate T-cell homeostasis. Herein, we report the most relevant clinical and experimental evidences of a pathogenic role of B cells in idiopathic nephrotic syndrome. We further highlight similarities and differences between children and adults affected by non-genetic forms of the disease and discuss what needs to be investigated in order to define the exact mechanisms underlying the pathogenic role of B cells and to identify more tailored therapeutic approaches.

## Introduction

Idiopathic nephrotic syndrome (INS), which includes two main histological entities, minimal change disease (MCD) and primary focal segmental glomerulosclerosis (FSGS), is the most frequent glomerular disease in childhood, with an incidence of 1.2-16.9/100,000 children (1). It is characterized by an intense proteinuria associated with hypoalbuminemia and edema due to an alteration of the glomerular permeability barrier leading to podocyte foot-process effacement (1). The response to a standardized steroid-treatment is more predictive of the disease course than histological findings in children, and kidney biopsy is usually not performed in steroid-sensitive INS (SSNS) pediatric patients (1). However, when biopsy is performed, most children present MCD (2). In contrast, kidney biopsy, which is routinely performed at onset of nephrotic syndrome in adults, shows a very low incidence of INS (0.6–1.8 per 100,000 adults), with the majority of patients presenting other glomerular diseases, mainly membranous nephropathy (2). In adult patients, MCD is less frequent than FSGS (3). Similarly to children, steroid treatment is the first-line therapeutic approach also for adults. However, a more prolonged treatment is necessary to induce remission compared to pediatric forms (2, 4). The pathogenesis of INS is still not well elucidated, but the therapeutic approaches based on immunosuppressive agents largely support a key role of the immune system in steroid-sensitive forms of the disease (5). In these forms, the disruption of the glomerular barrier leading to podocyte cytoskeleton disorganization and subsequent proteinuria seems to be mediated by one or more circulating factors, derived from dysregulated immune cells (6). Nonetheless, until now, the identity of this (these) factor(s) remain(s) undetermined (5, 6). Moreover, both in MCD and in FSGS the renal biopsy shows little or no evidence of glomerular inflammation and of immunoglobulin or complement deposition. For more than 30 years, INS was considered as a T-cell mediated disease (7), and several preclinical and clinical studies largely supported this hypothesis (5). More recently, a potential role of B cells has emerged due to the therapeutic efficacy of the B-cell depleting anti-CD20 antibodies (rituximab and ofatumumab) in inducing and/or maintaining a prolonged remission in children with INS (8–11). Similar results from non-prospective randomized clinical trials suggest that B-cells depleting agents may be useful in adult patients with steroid dependent or frequently relapsing INS to reduce the relapse frequency and to maintain remission despite cessation or tapering of steroids and/or immunosuppressive drugs (3). In addition, previous findings show that INS may occur in association with non-Hodgkin lymphoproliferative disorders or Hodgkin lymphoma where tumor cells derive from mature B cells (12–14) and with Epstein Barr virus infection, which mainly targets B cells (15). Altogether, these findings provide additional evidence implicating B lymphocytes as a key immune cell type involved in INS pathogenesis.

**Figure 1:**
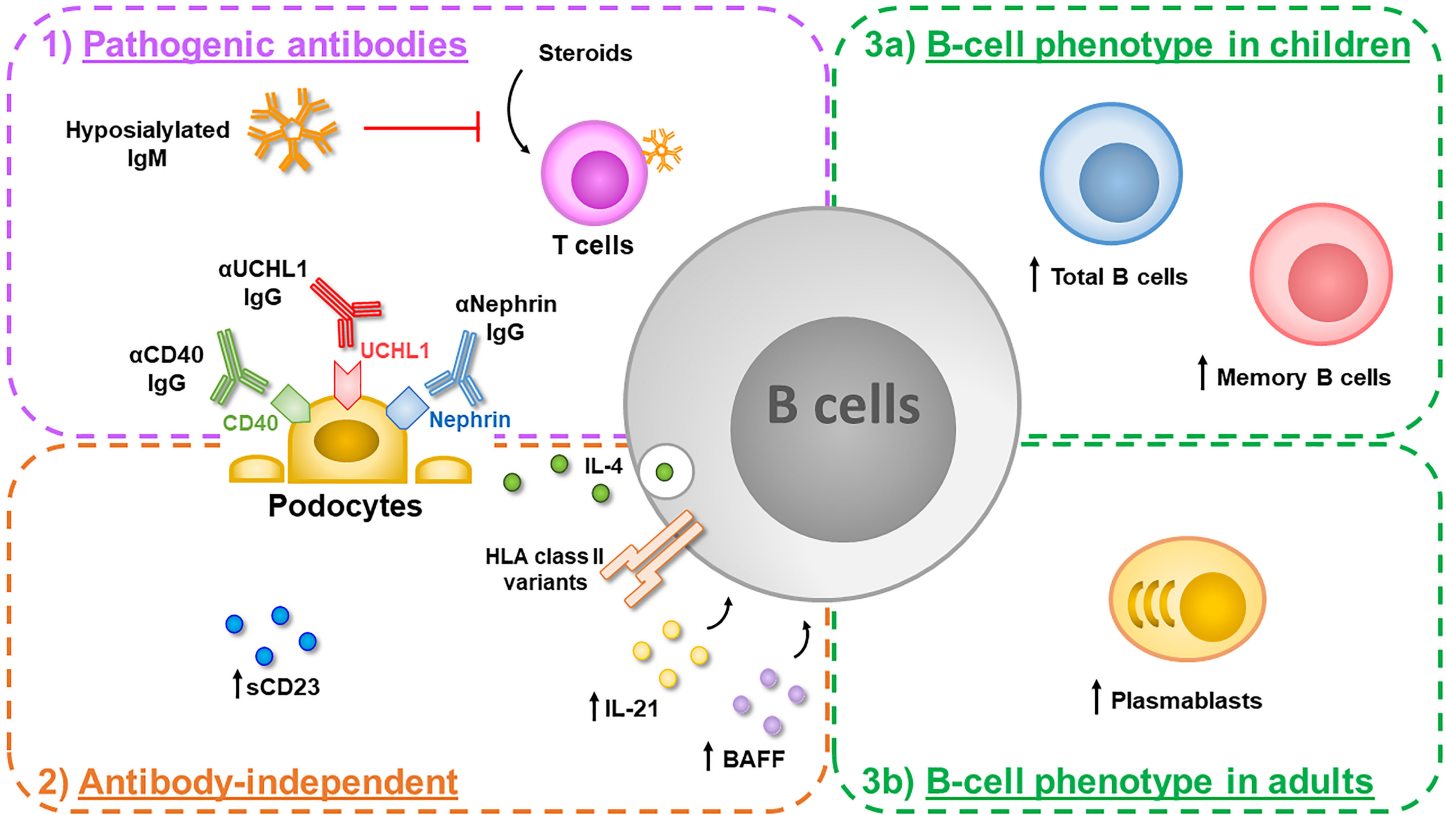
B cells can play a key role in the pathogenesis of idiopathic nephrotic syndrome (INS) thorough different mechanisms. 1) They can produce several potentially pathogenic antibodies targeting proteins constitutively expressed by podocytes such as anti-CD40 IgG, found in adults with recurrent focal segmental glomerulosclerosis (FSGS), anti-UCHL1 IgG, found in children with steroid-sensitive nephrotic syndrome (SSNS) and anti-nephrin IgG, found both in children and adults with minimal change disease (MCD). In addition, B cells produce hyposialylated IgM directed against T cells, which impair T-cell responses to steroid inhibition, in a subgroup of children with SSNS. 2) Activated B cells can also exert antibody-independent detrimental effects: an increased amount of serum CD23, a marker of B-cell activation, has been reported in pediatric SSNS and increased levels of serum B-cell activating factor (BAFF) and IL-21, which can contribute to B-cell activation, have been observed in adult MCD patients. In an experimental mouse model, IL-4 secreting B cells activated locally in the glomerulus induce podocyte effacement and proteinuria. In addition, specific risk alleles in the HLA region, which plays a crucial role in the antigen presentation to T cells, have been described in children with SSNS. 3) Alterations in B-cell homeostasis have been also observed during the active phase of the disease, with increased circulating levels of total B cells and memory B cells in children with SSNS (3a), and increased circulating levels of plasmablasts in adults with MCD (3b).

In this review, we describe phenotype and function of B cells and report the most relevant clinical and experimental indications of their potential role in the pathogenesis of INS. Current knowledge regarding potential effects exerted by B lymphocytes in INS pathogenesis is summarized in **Figure 1**.

## B-Cell Phenotype and Function

B cells are a heterogeneous population composed by different subsets and the physiological distribution of these subpopulations is age-dependent (16). During infancy, the peripheral B-cell pool is predominantly characterized by transitional B cells, which migrate from the bone marrow to the periphery where they acquire a naïve/mature phenotype. Through the circulation into secondary lymphoid organs, they encounter antigens that can induce their activation and differentiation into plasmablasts, which are the precursors of antibody secreting plasma cells, or memory B cells, which can produce IgM or undergo toward an isotype switching to IgG-, IgA-, or IgE-secreting B cells. Circulating plasmablasts and plasma cells generally die after few days, but some long-lived plasma cells return to the bone marrow and maintain antibody production independently of antigen exposure. From infancy to adulthood, the circulating levels of transitional, mature-naïve, memory B cells and plasmablasts/plasma cells progressively decrease, reaching a relatively stable number in adulthood (16).

Beside their ability in producing a large variety of antibodies that can protect from infections - the so called “humoral immunity” - B cells can also modify the homeostasis of specific tissues by producing immunoregulatory or pro-inflammatory cytokines and by expressing surface molecules able to present antigens which can in turn activate circulating T cells (17, 18). In particular, regulatory B cells produce immunomodulatory cytokines such as IL-10 and IL-35, which can dampen immune responses in pathological and transplant settings (18, 19). In parallel, B cells are able to directly or indirectly promote the activation of effector T cells by secreting pro-inflammatory cytokines such as IFN-gamma, TNF-alpha, IL-6 and IL-17 (20). During activation, B cells also express molecules such as HLA class II, CD40, CD80 and CD86, which stabilize the interactions between B and T cells and can sustain T-cell activation in autoimmune and auto-inflammatory diseases (17, 18, 21).

## Pathogenic Antibodies in INS

In contrast to the vast majority of the glomerular diseases, INS is usually not considered an antibody-mediated disease due to the lack of immunoglobulin deposits in kidney biopsies (22). Of note, glomerular IgM deposition, which can be observed in some forms of FSGS, seems to derive from a binding of IgM (together with activated complement factors) to epitopes that are exposed after injury of glomerular cells, suggesting that IgM may contribute to disease progression more than representing the causative factor (23, 24). However, a pathogenic role of circulating antibodies in INS has been hypothesized since 1998, when Dantal et al. showed that a permeability factor inducing albuminuria could be or could be bind to an immunoglobulin (25). In addition, an imbalance between serum IgG and serum IgM that sometimes persists during remission of the disease has been reported, suggesting a dysregulated immunoglobulin metabolism in INS (26, 27). More recently, potentially pathogenic IgG directed against surface podocyte proteins have been described in adult and pediatric INS (28–30). In particular, increased serum levels of anti-CD40 IgG were observed in adults with recurrent FSGS. These antibodies were able to cause a disruption of actin cytoskeleton of *in vitro* cultured podocytes and to induce proteinuria (in the presence of recombinant soluble urokinase plasminogen activator receptor) in injected mice (28). Unfortunately, the presence of glomerular IgG deposition following anti-CD40 injection was not evaluated in this study. In 2018, Jamin et colleagues reported increased serum levels of anti-UCHL1 IgG in children with SSNS (29). Anti-UCHL1 antibodies were able to cause a detachment of *in vitro* cultured podocytes and to induce an intense proteinuria when injected into mice (29). Interestingly, a pathological finding of MCD without glomerular IgG deposits was observed in injected mice and authors hypothesized that the binding of anti-UCHL1 IgG to surface UCHL1 could lead to podocyte detachment, explaining the absence of IgG deposits in INS kidney biopsies (29). Additional studies are needed to verify this intriguing hypothesis and to validate the real pathogenic role of the described anti-podocyte IgG. A more recent study showed that autoantibodies targeting nephrin, the major transmembrane protein of the podocyte slit diaphragm that links the interdigitating foot processes from neighboring podocytes, may be found in 29% of patients (both in children and adults) with biopsy-proven MCD (30). Since IgG deposits in kidney biopsy is an ultra-rare finding in kidney biopsy from patients with INS, the accurate role of these potential pathogenic antibodies remains to be determined. Nevertheless, this finding, highlighting that mechanisms underlying INS pathogenesis may involve autoimmune processes, deeply modifies our knowledge about this glomerular disease. Additional studies investigating the disrupting ability of the identified INS-associated autoantibodies, purified by sera or supernatants of *ex-vivo* isolated B-cell subsets, could be performed using the novel “glomerulus-on-a-chip” technology (31). This artificial platform mimics the glomerular permeability barrier composed of human podocytes, glomerular endothelial cells and extracellular matrix resembling the glomerular basement membrane (31).

In contrast to antibodies directed against podocytes, B cells could also exert detrimental effects by producing antibodies that can indirectly alter the immune homeostasis of INS patients. In this regard, a recent study reports the production of hyposialylated IgM directed against T cells, which inversely correlates with the extent of the response to steroid treatment, in a subgroup of SSNS pediatric patients (32). This study demonstrates that sialylated IgM targeting T cells are rapidly internalized and inhibit T-cell activation *in vitro*. In contrast, hyposialylated IgM, which can also target T cells, remain on the cell surface after binding but fail to exert inhibitory effects. More importantly, the binding of hyposialylated IgM renders T cells unresponsive to steroid inhibition and favors the secretion of podocyte damaging factors from activated T cells (32). These observations suggest that hyposialylated IgM could sustain T-cell activation induced by external triggers such as infections or atopy and could reduce T-cell response to steroid inhibition. Additional studies are needed to define the mechanism responsible of the production of hyposialylated IgM and to investigate whether immunosuppressive agents other than steroids can revert this phenomenon.

## Antibody-Independent Role of B Cells in INS

The potential role of T-cell derived cytokines in the pathogenesis of INS has been largely investigated, with contrasting results (6, 33). Among them, an increased production of IL-13 and TNF-alpha seems to be the most relevant and reproducible in INS (6, 33). In contrast, the role of B-cell derived cytokines has been poorly investigated. Of note, B cells are able to secrete both IL-13 and TNF-alpha, as well as other potentially pathogenic cytokines such as IL-4, IFN-gamma, IL-6 and IL-17 (20, 34). IL-13 and IL-4 cytokines are usually related to atopy, a condition that is widely associated with first or recurrent episodes of NS (35). In addition, an increased production of IL-4 from mononuclear cells isolated from MCD patients was associated to an increased expression of the B-cell surface CD23, a marker of B-cell activation, in the same patients (36). In 2003, increased levels of soluble CD23, in parallel with increased levels of soluble CD25 (a marker of T-cell activation), were also observed in SSNS pediatric patients in relapse, suggesting a potential role of B cells in sustaining T-cell stimulation (37). However, contrasting results on the amount of serum IL-4 in SSNS patients were reported during years (33). Of note, more than a circulating production of specific cytokines, a local secretion at glomerular level could also be pathogenically relevant in INS, since glomerular infiltration of T and B cells has been reported, especially in FSGS (38). In accordance with this hypothesis, Kim et colleagues elegantly demonstrated that IL-4-secreting B cells activated locally in the glomerulus can induce podocyte effacement and proteinuria in an experimental mouse model (39). The detrimental effects exerted by IL-4 on cultured human podocytes are currently being investigated in order to define its involvement in the pathogenesis of INS (40). Since B cells can secrete several immunoregulatory or pro-inflammatory cytokines, a local production of other B-cell derived cytokines could exert comparable or additional pathogenic effects. A recent report describes how IL-6 can alter the integrity of the glomerular permeability barrier and can derange actin cytoskeleton of *in vitro* treated podocytes, which constitutively express IL-6 receptor, suggesting a potential pathogenic role also for this cytokine (41).

In parallel with the production of specific cytokines, activated B cells could also directly contribute to T-cell stimulation by expressing surface activating molecules. SSNS is a heterogeneous disorder and the different forms of the disease are likely to be mediated by a complex interplay between the environment, the glomerular permeability barrier and the immune system (5). Despite the lack of a monogenic cause of the disease, genetic variants predisposing to develop SSNS following environmental triggers are emerging, as recently reviewed (42). Among all the identified SSNS-associated genetic variants, the strongest association was found in the HLA region, as identified by exome array and transethnic genome-wide association studies in large pediatric cohorts (43–46), supporting the role of an immune dysregulation in the antigen presentation machinery in SSNS forms (44). In agreement with this hypothesis, a recent study showed that INS relapses were associated with a decrease in T regulatory cells and IL-2 expression whereas remission phases under rituximab therapy were associated with a significant decrease in invariant natural killer T cells and CD4^-^CD8^-^ T-cells expressing the invariant Vα24 chain T-cells. These observations suggest that B-cell depleting agents may interfere in T cell-B cell cooperation during the course of the disease (47).

Additional studies should be performed to investigate the B-cell specific expression of activated molecules and their direct interaction with T cells in INS patients.

## B-Cell Phenotype in INS

In 2004, the casual observation of a sustained NS remission in a boy treated with rituximab for his recurrent idiopathic thrombocytopenic purpura proves for the first time the pathogenic role of B cells in INS (8). The successful use of B-cell depleting anti-CD20 monoclonal antibodies in maintaining long-lasting remission and allowing tapering of concomitant immunosuppressive treatment in both pediatric and adults INS patients has subsequently supported the hypothesis of a key role of B lymphocytes in the pathogenesis of INS (3, 9, 10). Interestingly, other immunosuppressive drugs frequently used for the treatment of INS, such as mycophenolate mofetil and calcineurin inhibitors, can target T cells but are also effective in inhibiting B-cell proliferation and immunoglobulin production, and can contribute to maintain remission following anti-CD20 treatment (5, 15, 48, 49). The amount of circulating B cells has been largely investigated in children with INS. **Table 1** summarizes the most relevant studies describing the B-cell phenotype in INS patients. High levels of B cells were already described in SSNS pediatric patients in 2002 (50). However, several subsequent studies reported conflicting results (51–53). Some of the observed discrepancies could be related to the immunosuppression received by most of the described patients before sampling, since the number of total B cells is determined by the proportion of different B-cell subsets that have differential sensitivities to immunosuppressive agents such as steroids, mycophenolate mofetil and calcineurin inhibitors (48). Prednisone treatment, for example, can exert long-lasting effects on different B-cell subsets also several months after interruption, as recently reported in children with SSNS (57). To avoid these confounding effects, the entire circulating B-cell repertoire has been characterized in a large cohort of SSNS pediatric patients at onset of disease before starting any immunosuppressive drug by Colucci et al. (54, 58). Increased levels of transitional and memory B cells, which together contributed to an increased amount of total B cells, were found. However, when SSNS patients treated with prednisone and one or two steroid-sparing agents were analyzed, transitional and mature/naïve B cells were mostly affected by immunosuppression, both in relapse and in remission, whilst memory B cells were found to be less sensitive to treatment (54). Similar results were also obtained by Ling and colleagues, who have recently demonstrated that increased levels of total, transitional and memory B cells can help in discriminating SSNS children from SRNS patients and that a reduced transitional/memory B-cell ratio is significantly predictive of SSNS recurrence (55, 56). Altogether, these results suggest that, among all the B-cell subpopulations, memory B cells play a key role in the pathogenesis of pediatric SSNS. Accordingly, the reappearance of memory B cells is highly effective in predicting relapse following anti-CD20 treatment in SSNS children (60–62).

**Table 1:** B-cell phenotype in idiopathic nephrotic syndrome patients.

	Type	Status	Treatment	B-cell subsets				
				Total CD19^+^	Transitional	Mature	Memory	Plasmablasts
**Children**								
Lama et al. (50)	SSNS	Relapse	Off therapy	↑	ND	ND	ND	ND
		Remission	Off therapy	↑	ND	ND	ND	ND
	SRNS	Relapse	Off therapy	↑	ND	ND	ND	ND
		Remission	Off therapy	↑	ND	ND	ND	ND
Kemper et al. (51)	SSNS	Relapse	Off therapy	↓	ND	ND	ND	ND
			On PDN	↑	ND	ND	ND	ND
		Remission	Off therapy	↓	ND	ND	ND	ND
			On PDN	↑	ND	ND	ND	ND
Lapillonne et al. (52)	SSNS	Relapse	Off therapy	=	ND	ND	ND	ND
		Remission	Off therapy	↓	ND	ND	ND	ND
Printza et al. (53)	SSNS	Onset	Untreated	↑	ND	ND	ND	ND
		Remission	On PDN	=	ND	ND	ND	ND
			After PDN discontinuation	=	ND	ND	ND	ND
Colucci et al. (54)	SSNS	Onset	Untreated	↑	↑	=	↑	ND
		Relapse	On PDN ± MMF ± CNIs	=	=	↓	↑	ND
		Remission	On PDN ± MMF ± CNIs	↓	↓	↓	=	ND
	Genetic SRNS	–	Untreated or on PDN or CNIs	=	=	=	=	ND
Ling et al. (55)	SSNS	Onset	Untreated	↑	↑	↓	↑	ND
		Relapse	On PDN	↑	=	↓	↑	ND
		Remission	On PDN	=	=	=	↑	ND
	SRNS	Onset	Untreated	=	=	↓	↑	ND
Ling et al. (56)	SSNS	Relapsing vs Non-relapsing	Untreated (onset) or on PDN (relapse and remission)	=*	↓*	=*	↑*	ND
Ling et al. (57)	SSNS	Onset	Untreated	↑	↑	=	=	ND
		Remission	On PDN	=	↓	=	↑	ND
			After PDN discontinuation	↓	↓	=	=	ND
Zotta et al. (58)	SSNS	Onset	Untreated	ND	ND	ND	ND	=
		Relapse	On PDN ± MMF ± CNIs	ND	ND	ND	ND	=
		Remission	On PDN ± MMF ± CNIs	ND	ND	ND	ND	=
**Adults**								
Oniszczuk et al. (59)	MCD	Relapse	Off therapy	=	=	=	=	↑
		Remission	Off therapy	=	=	=	=	=

SSNS, steroid-sensitive nephrotic syndrome; SRNS, steroid-resistant nephrotic syndrome; MCD, Minimal change disease.

Off therapy, patients without treatment for at least 6 months; untreated, patients at onset of the disease before starting prednisone treatment; PDN, prednisone; MMF, mycophenolate mofetil; CNIs, calcineurin inhibitors.

↑, increased values compared to age-matched healthy controls; ↓, reduced values compared to age-matched healthy controls; =, values comparable to those of age-matched healthy controls; ND, not determined.

*, Values compared between relapsing and non-relapsing patients.

In contrast to children, data regarding the distribution of circulating B cells in adult patients with INS at disease onset or during B-cell reconstitution after administration of anti-CD20 monoclonal antibodies are scarce. To our knowledge, only one study by Oniszczuk et al. showed that, among all the analyzed circulating B-cell subpopulations, plasmablasts were the only subset present at significantly higher levels during MCD relapses from untreated adult patients in comparison with patients in remission and with healthy controls (59). Of note, plasmablasts were also significantly increased in patients with biopsy-proven membranous nephropathy (an antibody-mediated glomerular disease characterized by an intense proteinuria, similar to that observed in MCD patients) (59). In this study, plasmablast levels during relapse were correlated with both lower albumin levels and higher proteinuria levels and were significantly associated to higher IgM levels and decreased IgG levels. In addition, an increased production of IL-21, IL-6 and B-cell activating factor (BAFF) was found in MCD patients with nephrotic-range proteinuria. Interestingly, the expansion of the plasmablast population may be partially explained by an increase of BAFF production, providing new evidence for the therapeutic use of anti-BAFF therapy (59). Of note, evaluation of plasmablast levels in children yielded conflicting results. Yang et al. reported higher levels of circulating plasmablasts in 94 children with new onset untreated INS patients, including both steroid-sensitive and steroid-resistant patients, compared to healthy controls (63). However, this increase in circulating plasmablasts was not confirmed in a subsequent study including only children with SSNS (58).

Altogether, these data suggest that alterations of the B-cell homeostasis observed in pediatric and adult INS patients are quite dissimilar, possibly due to the different maturity of the immune system between adults and children (16). This could also in part explain the distinct timing of response to steroid treatment in pediatric and adult patients with INS (2, 4), since prednisone differentially targets each B-cell subset (48).

Additional studies are needed to define the key pathogenic B-cell subset(s) in INS. A French multicenter trial is ongoing to test the efficacy of rituximab to maintain remission of a new-onset MCD episode in 98 adult patients who will either receive two doses of rituximab (375mg/m^2^ separated by 1 week) or a progressive tapering of prednisone doses (‘RIFIREINS’, NCT03970577, Rituximab From the FIRst Episode of Idiopathic Nephrotic Syndrome). The main objective of this study will be to demonstrate that the use of rituximab from the initial episode of MCD in adults may significantly reduce the risk of subsequent relapses and limit prolonged exposure to steroids without serious adverse events. Enrolled patients will be rigorously classified as steroid-sensitive, steroid-dependent or steroid-resistant INS based on their response to a standardized induction therapy with prednisone. In addition, this prospective trial will include an extensive B and T lymphocyte subpopulation monitoring, in order to investigate the potential close relationship between lymphocyte subpopulations and treatment response at different crucial timepoints and in different forms of the disease. The obtained results will help to improve our understanding of the role of lymphocyte subsets in the pathogenesis of INS in adulthood.

## Conclusion

The accurate molecular mechanisms underlying INS pathogenesis remain to be determined, but compelling data suggest that INS results from immune disorders leading to the release of circulating glomerular permeability factors, possibly T and/or B cell-derived, which in turn alter the glomerular filtration barrier and promote podocyte damages. The recent successful use of anti-CD20 monoclonal antibodies as supportive treatment of some forms of INS in children and adult patients provide additional evidence of a potential key role of B lymphocytes in the pathophysiological processes involved in this quite mysterious glomerular disease. Further studies are needed to elucidate the precise mechanisms by which and which B lymphocytes subpopulation could target the glomerular filtration barrier.

## Author Contributions

Conception and design: MC, JO, MV, and VA. Analysis of the data and preparation of the figure: MC and JO. Drafting and revision of the manuscript: MC, JO, MV, and VA. All authors contributed to the article and approved the submitted version.

## Conflict of Interest

The authors declare that the research was conducted in the absence of any commercial or financial relationships that could be construed as a potential conflict of interest.

## Publisher’s Note

All claims expressed in this article are solely those of the authors and do not necessarily represent those of their affiliated organizations, or those of the publisher, the editors and the reviewers. Any product that may be evaluated in this article, or claim that may be made by its manufacturer, is not guaranteed or endorsed by the publisher.
